# Safety and Efficacy of Shockwave Therapy for Myogenous Temporomandibular Disorders: Early Post-Treatment Results

**DOI:** 10.1016/j.identj.2025.103923

**Published:** 2025-09-23

**Authors:** Ka Wing Law, Andy Wai Kan Yeung, Yiu Yan Leung

**Affiliations:** a Oral and Maxillofacial Surgery, Faculty of Dentistry, The University of Hong Kong, Hong Kong, China; b Applied Oral Sciences & Community Dental Care, Faculty of Dentistry, The University of Hong Kong, Hong Kong, China

**Keywords:** Extracorporeal shockwave, Temporomandibular disorders, Myofascial pain, Facial pain, Temporomandibular joint

## Abstract

**Introduction and aims:**

To evaluate the early post-treatment safety and efficacy of extracorporeal shockwave therapy (ESWT) in the management of myogenous temporomandibular disorder (M-TMD).

**Methods:**

A randomized clinical trial following the CONSORT 2010 statement was conducted. Patients with M-TMD were recruited and randomly assigned in a 1:1 ratio into two groups: the ESWT Group and the Placebo Group. Three sessions of therapy were delivered. Pain score and jaw function were measured at pre-treatment baseline (T0), 1 week after each therapy session (T1, T2, T3), and 6 weeks (T4) and 3 months (T5) after the last session. Outcomes in pain score and jaw function were compared between the 2 groups.

**Results:**

Sixty-four patients (13 males, 51 females) with M-TMD were recruited with 33 patients in the ESWT group and 31 in the Placebo group. There were no differences between the 2 groups in the baseline pain score and jaw functions. The pain score in the ESWT group was remarkably lower than the Placebo group at the subsequent time points T1-T4. The overall mean decrease in pain in the ESWT group was 2.6 (*P* < .001). No significant difference was found in the jaw functions between the two groups. No significant complications or adverse effects have been reported.

**Conclusion:**

Extracorporeal shockwave could be a safe treatment option that provides more rapid and significant pain reduction.

**Clinical relevance:**

ESWT could be presented as an effective alternative treatment option in the management of M-TMD.

## Introduction

Temporomandibular joint disorder (TMD) represents collectively a heterogeneous group of orofacial pain conditions that have their origin in and emanate from the musculoskeletal structure of the masticatory system, consisting of the temporomandibular joint and the associated masticatory muscles.^1^ The Diagnostic Criteria for TMD (DC/TMD) divides the temporomandibular joint disorder into 2 main categories: pain-related TMD and intra-articular TMD.^2^ The pain-related TMD can be further subdivided into the myogenous component, namely myalgia, myofascial pain and myofascial pain with referral; and the arthrogenous component which is arthralgia. The intra-articular TMD encompasses various conditions including disc displacement with reduction, disc displacement with reduction with intermittent locking, disc displacement without reduction with limited opening, disc displacement without reduction without limited opening and degenerative joint disease. Patients can present with a variety of signs and symptoms including muscle soreness, joint pain, joint noise, hindered jaw movement and/or locking jaws.

Temporomandibular disorder has been reported to be a significant public health problem. It is the second most common musculoskeletal condition resulting in pain and disability, only ranking after lower back pain.^3^ When it comes to the orofacial region, temporomandibular disorder is the most common orofacial pain condition with 31% of the adult population and 11% of the children and adolescent population affected.^4^^,^^5^ In Hong Kong, a similar proportion was also reported, showing 1 out of 3 adults had symptoms of TMD.^6^

Contrary to arthrogenous TMD, the treatment modalities of M-TMD mainly encompass a range of conservative or minimally invasive measures. Pharmacological therapy is usually prescribed in the initial management of M-TMD.^7^ The most commonly employed classes of medication include non-steroidal anti-inflammatory drugs (NSAIDs), muscle relaxants and anti-depressants.^8^ However, pharmacological therapy is considered a short-term approach to TMD because of the potential untoward side effects of long-term use of medication.^9^ The long-term use of NSAIDs has been reported to cause gastrointestinal disturbance ranging from gastric pain to peptic ulcer.^10^ The most undesirable adverse effect with long-term use of the benzodiazepine class of muscle relaxants is potential physical dependence, even at a low therapeutic dose.^11^

In the past decades, occlusal splints have taken up a major role in the first-line treatment of M-TMD. Occlusal splints of various designs have been shown to alleviate the signs and symptoms of TMD.^12^, ^13^, ^14^, ^15^ An occlusal splint works by preventing the patients from achieving a maximal intercuspation^16^ but positioning the jaw in a way that allows the appropriate seating of the condyle in the centric relation^17^ and thus acquiring a new muscle and articular balance, reducing the influence of the occlusal interference.^18^ Physiotherapy has also been suggested to be an effective, low-cost management strategy to M-TMD. It consists of massage of the masticatory muscles, jaw-opening exercise, and heat pad application to the masticatory muscles. The efficacy has been reported to be comparable to occlusal splint therapy.^14^^,^^18^ It is important to note that the effectiveness of these conservative management strategies is largely dependent on patient’s compliance. The discomfort caused by wearing occlusal splints and the inability to adapt to the appliance, especially in mouth breathers, could adversely affect the treatment experience and compliance.

Other, less common therapeutic options exist, namely dry-needling,^19^ Botox injection^20^ and cognitive behavioral therapy.^21^, ^22^, ^23^ While some operators may have found favourable outcomes, some of these treatment modalities still lack quality evidence, which often presents with conflicting results, and this limits their use as a first-line treatment for M-TMD. For example, a systematic analysis has shown that low doses of botulinum toxin are effective in treating refractory myofascial pain associated with temporomandibular disorder.^24^ However, a Cochrane systematic review has suggested otherwise, reporting that the botulinum toxin was no better than the placebo.^25^

Extracorporeal shockwave therapy has gained popularity in recent years for the treatment of musculoskeletal problems. Shockwaves are rapid short bursts of acoustic energy that can propagate in a medium with a speed faster than sound, resulting in a disturbance of great amplitude. The exact mechanism of extracorporeal shockwave in bringing out its therapeutic outcome is not exactly understood. Yet, *in vivo* studies have demonstrated that extracorporeal shockwave therapy could be associated with upregulation of regenerative molecules, neoangiogenesis and modulation of anti-inflammatory mediators, which brings about tissue healing and pain relief effects.^26^, ^27^, ^28^, ^29^, ^30^ Promising results have been reported, and its application can be found in managing musculoskeletal diseases and sports-related tendinopathies.^31^^,^^32^ A randomised clinical trial studied the effect of extracorporeal shockwave therapy in managing the myofascial pain syndrome of upper trapezius muscles and found significant decrease in pain on the visual analog scale.^33^ A meta-analysis also showed that ESWT exhibited significant improvement in pain reduction compared to ultrasound treatment and placebo group, although there was no significant effect compared to conventional therapies.^34^ Compared to the multitude of evidence in the orthopedic and physiotherapy literature regarding the efficacy and application of extracorporeal shockwave, evidence with regard to the application of extracorporeal shockwave in masticatory muscles for the management of M-TMD remains scarce. To date, it is unknown whether ESWT is effective in treating M-TMD, and there are no data on its safety for this application in the head and neck region.

This randomised clinical trial therefore aimed to fill the knowledge gap in the literature by assessing the safety and efficacy of extracorporeal shockwave in the management of M-TMD.

## Materials and methods

This was the early post-operative findings of a randomised clinical trial to compare Extracorporeal Shockwave Therapy (ESWT) versus placebo in the management of M-TMD. The study protocol and the pilot data of this new treatment for M-TMD have been presented by our team previously.^35^ The trial design followed the CONSORT 2010 statement. Ethical approval from the Institutional Review Board of the University of Hong Kong / Hospital Authority Hong Kong West Cluster was obtained prior to the start of this study (HKU / HA HKW IRB: UW-20-704). Upon agreement to participate in the study, subjects were required to provide written informed consent.

### Study design

The study was a double-blinded (patients and operator) parallel-grouped clinical trial with balanced randomization (1:1)

### Participants

Patients who presented to the Division of Oral and Maxillofacial Surgery, Faculty of Dentistry, The University of Hong Kong with a clinical diagnosis of myogenous temporomandibular disorders were invited to participate in the study. The clinical diagnoses of masticatory muscle pain, joint pain, and intra-articular joint conditions were made based on the Diagnostic Criteria for Temporomandibular Disorders (DC/TMD).^2^ Patients were included in the trial if they were at least 16 years of age; had pain in the masticatory muscles, headache attributed to TMDs, with or without limited mouth opening; and had pain in the temporomandibular joint (TMJ).

Patients were excluded from the recruitment if they presented with the following conditions: (1) pain in the TMJ only and not involving muscles of mastication; (2) active infection in the TMJ region; (3) systemic rheumatic diseases; (4) significant systemic diseases; (5) craniofacial syndromes; and (6) previous operations in the TMJ.

Upon the diagnosis of M-TMD after the initial assessment, a prescription for a 2-week course of ibuprofen (400 mg TDS) or paracetamol (500 mg QID) if the patients were allergic to NSAIDs was provided. The patients were recruited into the study if their symptoms persisted despite the initial course of analgesics.

### Randomisation

A simple randomisation was used in this study. Recruited patients were randomly assigned to 1 of the 2 study groups, Extracorporeal Shockwave Therapy (ESWT) group and the Placebo group. By using a computer program, a randomisation table was generated. The allocation sequence was concealed in sequentially numbered, opaque, sealed envelopes and was kept by the research assistant. Upon obtaining the study consent from the participants, the research assistant opened the sealed envelope containing the allocation sequence, and the same person was responsible for setting up the handpiece stand-off according to the respective groups, blinding the patient and the operator who was providing the treatment.

### Interventions

The two arms of the intervention were ESWT versus placebo. After the initial clinical assessment and allocation procedure, patients were treated accordingly based on their allocated groups to which both the patient and the operator were blinded.

### Extracorporeal shockwave therapy

In the ESWT group, stand-off II was connected to the Focused SEPIA® handpiece (DUOLITH® SD1T-TOP, Storz Medical). At each session 500 pulses of focused extracorporeal shockwaves were delivered to the involved masseter muscle at a setting of 0.15 mJ/mm^2^ energy and 6 Hz frequency as per manufacturer’s recommendations for craniomandibular dysfunction. One session of therapy per week and a total of 3 sessions were provided.

### Placebo

In the Placebo group, a placebo stand-off containing air that blocked the transmission of shockwave energy was connected to the handpiece. The same procedure as in the ESWT group was carried out with the same weekly treatment interval, and a total of 3 sessions were provided.

### Outcome Measures

The primary outcome of the randomised clinical trial was pain symptoms 6 weeks after the last shockwave session, as measured by a numerical rating scale (NRS). The secondary outcomes were the subjects’ TMJ functions in terms of mouth opening, protrusion and lateral excursions.

### Data collection

One single-blinded assessor not aware of the grouping of the patients carried out the following clinical assessments.

### Pain assessment

Preoperative measurement of pain symptoms was made on a 0 to 10 numerical rating scale (NRS) within the Graded Chronic Pain Scale,^36^ with 0 indicating no pain and 10 indicating maximal pain. Post-operative measurement of pain symptoms was done with an NRS 1 week after each treatment session and at 6 weeks and 3 months after the last treatment session. The timepoints of assessments were: T0: Pre-treatment; T1: 1 week after the first session; T2: 1 week after the second session; T3: 1 week after the third session; T4: 6 weeks after the third session; and T5: 3 months after the third session.

### TMJ function assessment

Unassisted maximal mouth opening (MMO) was measured by the vertical interincisal distance upon maximal opening minus the overbite. Protrusion was measured by the horizontal distance between the upper and lower incisal edges upon protrusive movement minus the overjet. Lateral excursions were measured by the horizontal distance between the midlines of the upper and lower central incisors upon left and right lateral excursive movements minus the midline deviation of the lower central incisors with reference to the upper incisors at rest. Assessments of TMJ function were performed pre-treatment, at 1 week after each treatment session and at 6 weeks and 3 months after the last treatment session. MMO, lateral excursions to the left and the right, and protrusion were recorded in millimetre.

### Complications

Details regarding complications that occurred in the peri-treatment period were recorded.

### Sample size calculation

The primary outcome measure was pain, measured by NRS, at 6 weeks post-treatment. Lacking previous studies, we grouped the patients into either pain or no pain, with 100% of the pre-treatment subjects presenting with pain, for sample size calculation purposes. We hypothesised that there would be a 50% and 20% reduction in the proportion presenting with pain post-operatively in the ESWT and Placebo group, respectively. To see this 30% difference with 80% power, we would need 39 patients per group. Considering the potential patient dropout, we planned to recruit 45 patients into each group so 90 patients in total would be needed. This study presented the findings of the early post-operative results for those patients who were reviewed for 3 months. Sample size calculation was performed using the online sample size calculator ‘Inference for Proportions: Comparing Two Independent Sample’, available at: https://www.stat.ubc.ca/~rollin/stats/ssize/b2.html

### Statistical methods

Statistical analysis was performed using SPSS 28 software (SPSS Inc.). The independent *t*-test and chi-square test were used for the comparison of patient demographic data. Repeated measures analysis of variance (ANOVA) was employed to compare the changes over the 6 time points. *Post hoc* tests with Bonferroni correction were done using multiple paired-sample tests for the comparison of 2 time points within the same group. Between-group comparison at the same time point was performed with an independent *t*-test. The statistical significance level was set at *P* < .05.

## Results

### Subject enrollment

**Fig. 1:**
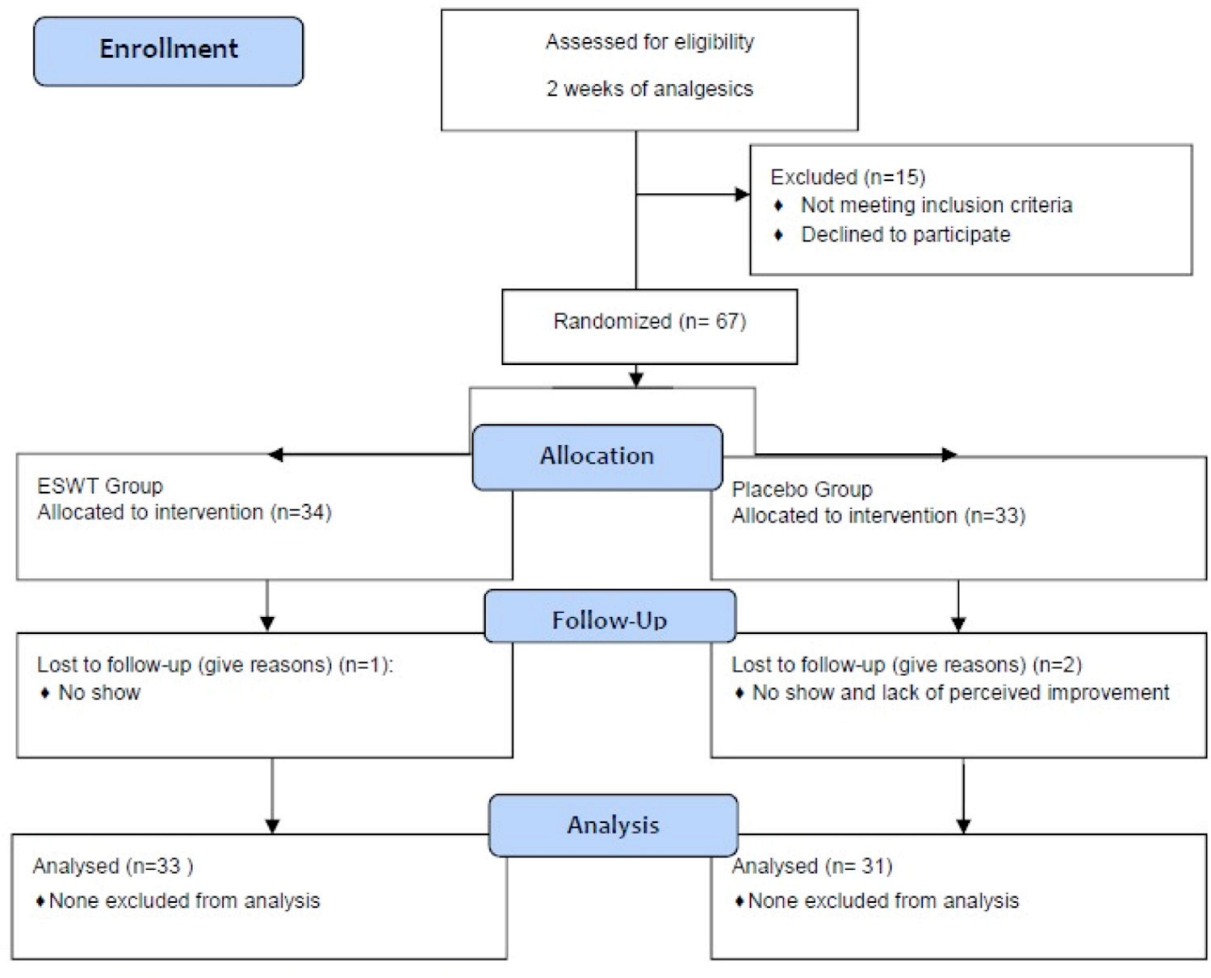
CONSORT study flow chart (Version 2010).

A total of 67 patients whose symptoms of m-TMD were not resolved by analgesics including NSAIDs or paracetamol were recruited into the study. The recruited patients were enrolled in the randomisation process after providing informed consent. Among the recruited subjects, 34 were allocated to the ESWT group and 33 were allocated to the Placebo group. A total of 3 patients dropped out of the study, 1 patient from the ESWT group and 2 from the Placebo group. Subsequently, 33 patients in the ESWT group and 31 patients in the Placebo group completed the 3-month follow-up period and were subjected to final statistical analysis (Figure 1).

### Demographics

The demographic information is summarised in Table 1. There was no difference in the proportion of male-to-female subjects between the two groups (*P* = .153). There was also no difference in the mean age of patients in the ESWT group (44.2 years ± 19.0 years) and the Placebo group (48.7 years ± 17.6 years) (*P* = .327). In terms of the clinical diagnosis, myalgia was the majority in both groups, with a proportion of 84.8% in the ESWT group (*n* = 28) and 83.9% in the Placebo group (*n* = 26). More than half of the subjects in both groups presented with arthralgia. There were 30.3% and 48.4% of the patients in the ESWT group and the Placebo group, respectively, who had disc displacement with reduction. The mean onset time of symptoms was 16.1 months for the ESWT group and 26.6 months for the Placebo group. There were no statistically differences in the clinical characteristics between the ESWT group and Placebo group (*P* > .05).

**Table 1:** Demography and temporomandibular dysfunction diagnoses of the subjects.

	ESWT group	Placebo group	*P*
Sample size	33	31	
Gender			.153
Male, % (*n*)	27.3% (9)	12.9% (4)	
Female, % (*n*)	72.7% (24)	87.1% (27)	
Age, years (SD)	44.2 ± 19.0	48.7 ± 17.6	.327
Onset of symptoms, months (SD)	16.1 ± 21.8	26.6 ± 44.2	.121
Diagnosis of painful conditions			
Muscle pain conditions			.914
Myalgia, % (*n*)	84.8% (28)	83.9% (26)	
Myofascial Pain with referral, % (*n*)	15.2% (5)	16.1% (5)	
Joint pain conditions			.332
Arthralgia, % (*n*)	66.7% (22)	54.8% (17)	
Diagnosis of intra-articular conditions			.325
Disc displacement with reduction, % (*n*)	30.3% (10)	48.4% (15)	
Disc displacement without reduction with limited mouth opening, % (*n*)	15.2% (5)	9.7% (3)	

SD, standard deviation.

### Pain

Table 2 summarises the pain score at each time point for each group and the difference in pain scores between each group at each time point. The initial pain scores at T0 baseline were 5.6 ± 1.6 for the ESWT group and 5.9 ± 1.4 for the Placebo group. There was no statistically difference in the baseline pain score between the two groups (*P* = .393).

**Table 2:** Pain score (NRS): Between-group comparison.

Time point	ESWT group, mm (SD)	Placebo group, mm (SD)	*P*
T0: Pre-treatment	5.6 ± 1.6	5.9 ± 1.4	.393
T1: 1 week after first session	4.2 ± 2.1	5.8 ± 2.0	.003*
T2: 1 week after second session	3.4 ± 2.2	5.0 ± 2.0	.004*
T3: 1 week after third session	3.4 ± 2.3	4.7 ± 2.2	.027*
T4: 6 weeks after third session	3.1 ± 2.2	4.3 ± 2.2	.028*
T5: 3 months after third session	3.0 ± 2.2	4.1 ± 2.4	.059

⁎ Statistical significance. SD, standard deviation.

**Fig. 2:**
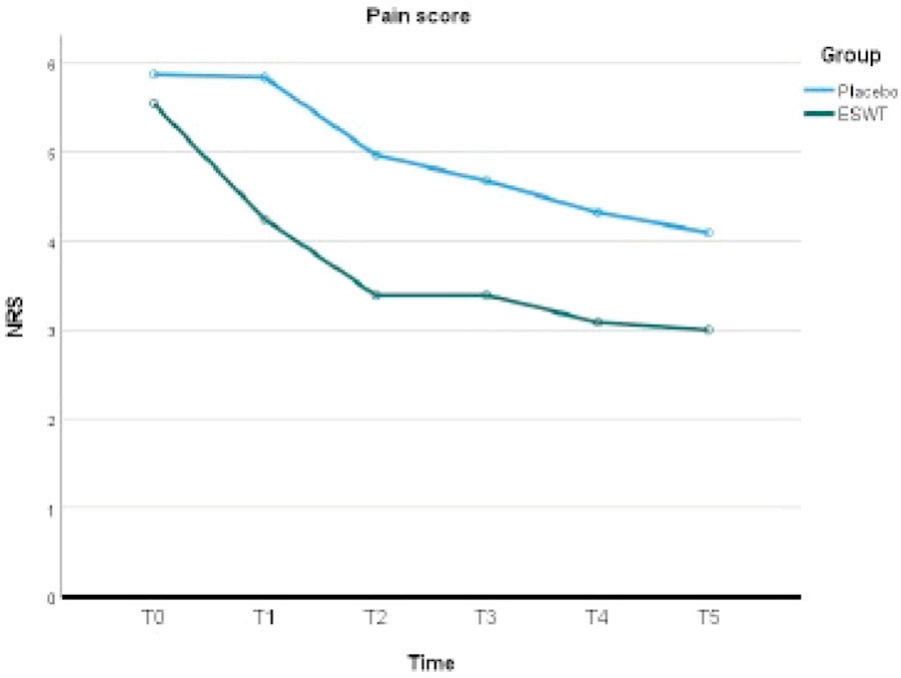
Pain score of ESWT and placebo.

The ESWT group demonstrated a significantly lower pain score with statistical significance compared to the Placebo group at T1 (*P* = .003), T2 (*P* = .004), T3 (*P* = 0.027) and T4 (*P* = .028). There was no significant difference between the two groups at T5 (3 months after the third session) (*P* = .059). For pain reduction over the course of therapy, the ESWT group showed the most significant reduction in pain over the period between T0 and T1 (Mean difference (MD) = 1.3, Standard Error (SE) = 0.32, *P* = .002) and between T1 and T2 (MD = 0.85, SE = 0.21, *P* = .002). There was a continuous reduction in pain during the review period. In the Placebo group, there was also a reduction in pain over the course of the review period. Overall, the ESWT group achieved a significant reduction in pain with a mean difference of 2.6 (SE = 0.42, *P* < .001) over the period of 3 months whereas the Placebo group showed a smaller degree of reduction (MD = 1.8, SE = 0.43, *P* = .002). Table 3 demonstrates the reduction in pain score within each group. The trend of pain reduction is illustrated in Figure 2.

**Table 3:** Pain score (NRS): Within-group comparison.

ESWT group			
Time points	MD (mm)	SE	*P*
T0–T1	1.3	0.32	.002*
T1–T2	0.85	0.21	.002*
T2–T3	0.00	0.22	1.000
T3–T4	0.30	0.21	1.000
T4–T5	0.09	0.28	1.000
T0–T5	2.6	0.42	<.001*


Group 2: Placebo			
Time points	MD (mm)	SE	p-value
T0–T1	0.03	0.33	1.000
T1–T2	0.87	0.21	.002*
T2–T3	0.29	0.23	1.000
T3–T4	0.36	0.22	1.000
T4–T5	0.23	0.29	1.000
T0–T5	1.8	0.43	.002*

⁎ Statistical significance after Bonferroni correction. MD, mean difference; SE, standard error.

### Maximal mouth opening

**Fig. 3:**
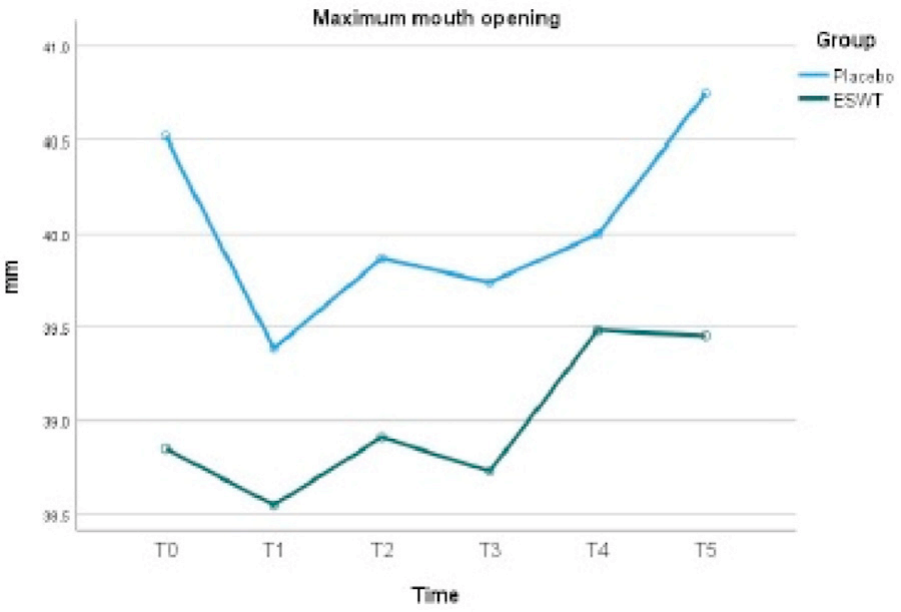
Maximal mouth opening of ESWT and placebo.

Table 4 summarizes the mean maximal mouth opening (MMO) in the ESWT and Placebo groups and their difference at each time point. At baseline, the maximal mouth openings in the ESWT group and the Placebo group were 38.9 mm ± 9.1 mm and 40.5 mm ± 6.2 mm, respectively. There was no statistical difference (*P* = .395). There were no statistical differences in the maximal mouth opening between the two groups at all time points. At T5 (3 months after the third session), the mean maximal mouth openings in the ESWT group and the Placebo group were 39.5 mm ± 8.2 mm and 40.7 mm ± 7.4 mm, respectively. Table 5 demonstrates the mean difference in maximal mouth opening across different time points within the ESWT group and the Placebo group. Figure 3 illustrates the trend of change in maximal mouth opening for each group.

**Table 4:** Maximal mouth opening: Between-group comparison.

Time point	ESWT group, mm (SD)	Placebo group, mm (SD)	*P*
T0: Pre-treatment	38.9 ± 9.1	40.5 ± 6.2	.395
T1: 1 week after first session	38.6 ± 9.0	39.4 ± 6.4	.670
T2: 1 week after second session	38.9 ± 8.3	39.9 ± 6.7	.612
T3: 1 week after third session	38.7 ± 8.4	39.7 ± 6.7	.595
T4: 6 weeks after third session	39.5 ± 8.9	40.0 ± 5.6	.783
T5: 3 months after third session	39.5 ± 8.2	40.7 ± 7.4	.515

SD, standard deviation.

**Table 5:** Maximal mouth opening: Within-group comparison.

ESWT group			
Time points	MD (mm)	SE	*P*
T0–T1	0.30	0.77	1.000
T1–T2	–0.36	0.55	1.000
T2–T3	–0.18	0.45	1.000
T3–T4	–0.76	0.54	1.000
T4–T5	0.03	0.59	1.000
T0–T5	–0.61	0.88	1.000


Placebo group			
Time points	MD (mm)	SE	p-value
T0–T1	1.1	0.79	1.000
T1–T2	–0.48	0.57	1.000
T2–T3	0.14	0.46	1.000
T3–T4	–0.26	0.56	1.000
T4–T5	–0.74	0.61	1.000
T0–T5	–0.23	0.91	1.000

MD, mean difference; SE, standard error.

### Lateral excursions

The baseline extent of lateral excursions to the left side reported in the ESWT and Placebo groups were 6.3 mm ± 3.0 mm and 6.4 mm ± 2.1 mm, respectively, and the extent of lateral excursions to the right side was 6.2 mm ± 2.7 mm in the ESWT group and 6.1 mm ± 2.1 mm in the Placebo group. No statistically differences were found between the 2 groups in all the review time points. The mean lateral excursions and the mean differences between the 2 groups at each time point are presented in Table 6.

**Table 6:** Lateral excursions: Between-group comparison.

Time points	Laterality	ESWT group, mm (SD)	Placebo group, mm (SD)	*P*
T0: Pre-treatment	R	6.2 ± 2.7	6.1 ± 2.1	.847
	L	6.3 ± 3.0	6.4 ± 2.1	.899
T1: 1 week after first session	R	6.3 ± 2.7	6.6 ± 1.8	.672
	L	6.2 ± 2.6	6.3 ± 2.1	.817
T2: 1 week after second session	R	6.9 ± 2.3	6.4 ± 2.2	.365
	L	6.6 ± 2.0	6.4 ± 2.2	.813
T3: 1 week after third session	R	7.0 ± 2.3	6.6 ± 2.2	.494
	L	6.7 ± 2.4	6.7 ± 2.0	.982
T4: 6 weeks after third session	R	6.9 ± 2.3	6.9 ± 2.0	.989
	L	6.8 ± 2.1	6.7 ± 2.1	.887
T5: 3 months after third session	R	6.9 ± 2.3	7.3 ± 2.4	.524
	L	6.8 ± 2.1	7.0 ± 2.3	.658

Laterality: R = right, L = left. SD, standard deviation.

### Protrusion

**Fig. 4:**
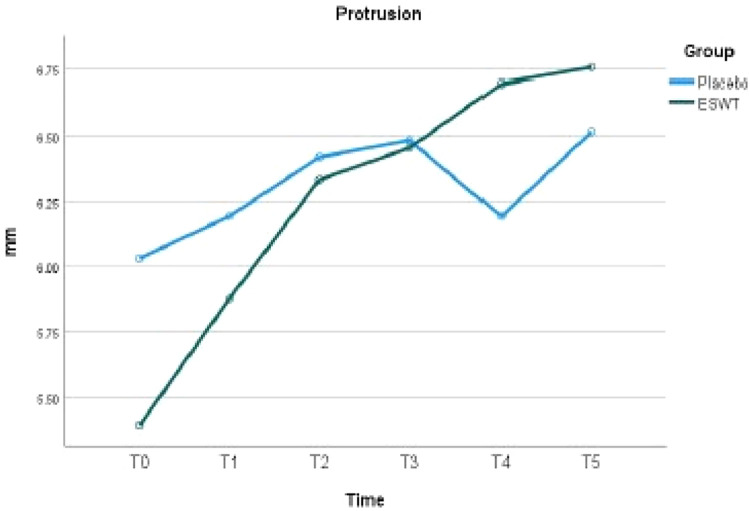
Protrusion in the ESWT and Placebo groups.

The baseline mean protrusion was 5.4 mm ± 2.9 mm in the ESWT group and 6.0 mm ± 2.3 mm in the Placebo group. There was no statistical difference between the 2 groups at baseline (*P* = .336). Statistically significant differences were not found at all the review time points between the 2 groups. The comparison of protrusion between groups at each time point is summarised in Table 7. As demonstrated in Table 8, it was found that the ESWT group showed a significant improvement in protrusion to 6.8 mm ± 1.8 mm at 3 months after treatment (*P* = .011). There was no significant improvement in protrusion in the Placebo group (Figure 4).

**Table 7:** Protrusion: Between-group comparison.

Time point	ESWT group, mm (SD)	Placebo group, mm (SD)	*P*
T0: Pre-operative	5.4 ± 2.9	6.0 ± 2.3	.336
T1: 1 week after first session	5.9 ± 2.5	6.2 ± 2.2	.590
T2: 1 week after second session	6.3 ± 2.4	6.4 ± 1.9	.872
T3: 1 week after third session	6.5 ± 2.6	6.5 ± 1.9	.959
T4: 6 weeks after third session	6.7 ± 2.2	6.2 ± 2.1	.359
T5: 3 months after third session	6.8 ± 1.8	6.5 ± 2.0	.612

SD, standard deviation.

**Table 8:** Maximum mouth opening: Within-group comparison.

Group 1: ESWT			
Time points	MD (mm)	SE	*P*
T0–T1	–0.49	0.30	1.000
T1–T2	–0.46	0.29	1.000
T2–T3	–0.12	0.25	1.000
T3–T4	–0.24	0.23	1.000
T4–T5	–0.06	0.26	1.000
T0–T5	–1.4	0.38	.011*


Group 2: Placebo			
Time points	MD (mm)	SE	*P*
T0–T1	–0.16	0.31	1.000
T1–T2	–0.23	0.30	1.000
T2–T3	–0.07	0.26	1.000
T3–T4	0.29	0.24	1.000
T4–T5	–0.32	0.26	1.000
T0–T5	–0.48	0.40	1.000

⁎ Statistical significance after Bonferroni correction. MD, mean difference; SE, standard error.

### Complications / Adverse effect

No major complications or adverse effects were reported in either group. No significant discomfort was reported by any subjects during the sessions. There was no facial paralysis, paraesthesia, haematoma or swelling reported. One of the subjects in the ESWT group complained of tinnitus in bilateral ears after the first session of ESWT, which resolved within a week.

## Discussion

This randomised controlled trial presented the early post-treatment findings of the clinical outcomes and safety of extracorporeal shockwave therapy as a new treatment for M-TMD when compared to placebo. The key findings of the study are as follows: (1) ESWT is effective in reducing pain in patients with M-TMD in the early post-treatment period when compared to placebo; (2) there are no major complications or adverse effects of using ESWT for the treatment of M-TMD in the masseter region; and (3) no significant difference was found in the maximum mouth opening, lateral excursion and protrusion in the two groups in the early post-treatment period.

Shockwaves are rapid, short, and distinct fluctuations of acoustic energy from a positive to a negative phase.^37^ They cause a propagating disturbance of great amplitude which travels in a medium faster than the speed of sound. The two major shockwave modalities employed in the therapeutic setting are focused and radial shockwaves. Focused shockwaves concentrate the acoustic energy on a well-defined point of the target tissue. Radial shockwaves, on the other hand, transfer a diverging acoustic energy with the highest energy on the skin surface and subsequently weaken as they propagate further into deeper tissues.^38^ The earliest application of extracorporeal shockwave was for treating urolithiasis.^39^ Its application was later extended to musculoskeletal diseases and sports-related overuse tendinopathies including non-union of pseudoarthrosis or fracture, calcific or non-calcific tenosynovitis of the shoulder, plantar fasciitis, lateral epicondylitis of the elbow and patellar tendinopathy.^31^^,^^32^

The exact mechanism of extracorporeal shockwave’s therapeutic outcome is not exactly understood. However, it has been hypothesised that there is a mechanotransduction process at the cellular level, in which reactive cells with mechanosensibility are able to transmit exogenous stimuli—that is, the acoustic impulse—into biological information resulting in a biological response.^37^ One of the biological responses observed is the increase in molecules associated with tissue regenerative processes such as the upregulation of vascular endothelial growth factor (VEGF),^26^^,^^27^ fibroblasts growth factor (FGF) and endothelial nitric oxide synthase (eNOS).^28^ On the tissue level, it has been shown that neoangiogenesis occurs with an increase in vascular density and local blood flow, and this property has also opened up a new perspective for managing chronic skin ulcers and non-healing wounds.^29^ An additional effect of extracorporeal shockwave is the modulation of anti-inflammatory mediators and endorphins, which contributes to its pain-relieving property.^30^ The success observed in the treatment of trapezius muscle trigger points has shed light on the potential of extracorporeal shockwave in managing myofascial pain syndrome.^33^^,^^34^

Results of the current study show that there was a reduction in the pain score in both the ESWT and placebo groups, but there was more significant pain reduction in the ESWT group in the early post-treatment period, showing a mean reduction of pain by 2.6 on the numerical rating scale. This is above the minimally clinically important difference as suggested by a clinical significance analysis study which concluded that women with TMD presented a large improvement in their general health status if they presented with a pain reduction of 1.9 cm on visual analog scales.^40^ The most pronounced reduction in pain was found during the first 2 weeks of therapy. Previous research of ESWT on treating TMD was generally weak and underpowered.^41^^,^^42^ Our randomised clinical trial, following a stringent study design and with a bigger sample size, offers strong evidence of the efficacy and safety of ESWT in treating specifically M-TMD.

It is noticeable that despite the pain reduction effect of ESWT found in this study, the proportion of patients in the ESWT group achieving complete pain resolution still remains low. Due to the multifactorial aetiology of this condition, relying on a single treatment modality for bringing about a pain-free state appears to be unrealistic. The historical account for M-TMD focused on structural disturbance and malalignment in the stomatognathic system. Early works from the prosthodontic and orthodontic literature concluded the role of occlusal vertical dimension, occlusal disharmony and neuromuscular imbalance in the development of TMD.^43^, ^44^, ^45^ There is also a novel insight into the role of masticatory muscle imbalance as a result of facial asymmetry and other forms of dentofacial deformity in relation to TMD symptoms.^46^, ^47^, ^48^

The explanation of TMD with a purely structural model has been proven incomplete due to the phenomenon that not all patients with a physical abnormality present with the same degree of clinical manifestation of TMD. In recent years the biopsychosocial aspect of TMD has gained much more emphasis.^49^^,^^50^ Some researchers have demonstrated that individuals with TMD symptoms showed a higher level of distress, depression, anxiety and somatisation.^51^^,^^52^ The close relationship between various psychological factors and TMD has also been concluded by a prospective cohort study of the Orofacial Pain: Prospective Evaluation and Risk Assessment (OPPERA), which reported that several premorbid psychological variables including measures of global and psychological somatic symptoms, perceived stress, previous life events and negative affectivity predicted the onset of TMD.^53^ Furthermore, the symptoms of pre-existing M-TMD have also been demonstrated to exacerbate during particular stressful times. Studies in the COVID-19 era have suggested that the impact of lockdown and social isolation during the pandemic is associated with the prevalence of depression, stress and the painful symptoms of TMD.^54^, ^55^, ^56^ It appears that TMD represents a condition involving the interplay between a biological problem and a psychological state in a social framework. Extracorporeal shockwave therapy could be used in combination with other treatment modalities to address the multitude of underlying biomechanical and psychosocial causes.

It is noteworthy that in the demographic characteristics of the current studied subject population, the mean period of symptom onset to treatment ranged from 16 to 26 months. The chronic nature of pain could have contributed to the failure of complete eradication of pain by extracorporeal shockwaves alone. Chronic primary pain is defined as the persistence of pain for a duration of more than 3 months with associated significant emotional distress and/or functional disability, and with the pain not better accounted for by another condition.^57^ It has been proposed that the phenomenon of central sensitisation plays a role in the development of chronic primary pain.^58^ Central sensitisation refers to the central nervous system mechanisms which amplify the nociceptive signal, involving the dysregulation of inhibitory interneurons in the dorsal horn and the descending modulatory pathways, changes in the molecular expression of neurotransmitters and receptors and neuroplasticity of the central nervous system which refers to a continuous process of reorganisation of synaptic networks to perceived experiences to the environment.^59^ Central sensitisation and neuroplasticity could represent a basis for the transition of an acute state to a chronic pain condition that presents as an obstacle to the pain management.

One of the limitations of the current study is the heterogeneity of the recruited participants, which could be one of the attributing factors for the lack of significant improvement in terms of maximal mouth opening, protrusion and lateral excursion in the early post-treatment period. It has been acknowledged that TMD patients often present with a combination of myogenous and arthrogenous conditions including disc displacement without reduction with limited mouth opening. It is speculated that in some of the participants the myalgia was managed with ESWT but the intra-articular conditions remained unaddressed, which presented as one of the confounding factors. This highlights the importance of a multifaceted treatment strategy for managing TMD.

The current study presented the early results of a larger project which also aimed to investigate the long-term effect of ESWT in treating M-TMD patients. It is still unknown if the beneficial effect of ESWT could be long-lasting, and that could only be proven with longer follow-up. It is also unknown what the best frequency or dosage of the ESWT for treatment of M-TMD is, because the current setting is based on the location and thickness of the target muscle as recommended by the company.

The current study used a protocol with an energy output of 0.15 mJ/mm^2^ at 6 Hz frequency for 500 cycles at each session and a total of 3 sessions. A beneficial effect in pain reduction with the current protocol has been observed. This study, showing the short-term efficacy of ESWT, has formed a good foundation for future research to focus on discovering the optimal parameters of the treatment protocol in terms of the energy, cycles per session, number of sessions needed, modality of shockwave therapy, and combination of different treatment modalities to provide the maximal therapeutic outcome.

## Conclusion

This double-blinded randomised clinical trial has shown ESWT as effective in managing M-TMD in terms of pain reduction in the early post-treatment period, but there is a limited impact on TMJ function in terms of maximal mouth opening, protrusion and lateral excursion. No major complications or adverse effects were reported in using ESWT in treating M-TMD, and it is considered safe to use. Longer follow-up will be required to show the long-term outcome of this new treatment modality, as well as the optimal frequency and dosage of ESWT in treating M-TMD.

## Author Contributions

*Conceptualisation:* Law, Leung, Yeung

*Design:* Law, Leung, Yeung

*Data collection and analysis:* Law

*Writing—original draft:* Law

*Writing—revising and editing:* Law, Leung, Yeung

## Funding

This study was funded by the Health and Medical Research Fund, the Health Bureau, and the Government of the Hong Kong Special Administrative Region (Reference Number: 19200851).

## Conflict of interest

The authors declare that they have no known competing financial interests or personal relationships that could have appeared to influence the work reported in this paper. The author is an Associate Editor for this journal and was not involved in the editorial review or the decision to publish this article.
